# Aesthetic Pleasure versus Aesthetic Interest: The Two Routes to Aesthetic Liking

**DOI:** 10.3389/fpsyg.2017.00015

**Published:** 2017-01-30

**Authors:** Laura K. M. Graf, Jan R. Landwehr

**Affiliations:** Chair for Product Management and Marketing Communications, Goethe University FrankfurtFrankfurt am Main, Germany

**Keywords:** aesthetic liking, attractiveness, pleasure, interest, processing fluency, processing style

## Abstract

Although existing research has established that aesthetic pleasure and aesthetic interest are two distinct positive aesthetic responses, empirical research on aesthetic preferences usually considers only aesthetic liking to capture participants’ aesthetic response. This causes some fundamental contradictions in the literature; some studies find a positive relationship between easy-to-process stimulus characteristics and aesthetic liking, while others suggest a negative relationship. The present research addresses these empirical contradictions by investigating the dual character of aesthetic liking as manifested in both the pleasure and interest components. Based on the Pleasure-Interest Model of Aesthetic Liking (PIA Model; Graf and Landwehr, 2015), two studies investigated the formation of pleasure and interest and their relationship with aesthetic liking responses. Using abstract art as the stimuli, Study 1 employed a 3 (stimulus fluency: low, medium, high) × 2 (processing style: automatic, controlled) × 2 (aesthetic response: pleasure, interest) experimental design to examine the processing dynamics responsible for experiencing aesthetic pleasure versus aesthetic interest. We find that the effect of stimulus fluency on pleasure is mediated by a gut-level fluency experience. Stimulus fluency and interest, by contrast, are related through a process of disfluency reduction, such that disfluent stimuli that grow more fluent due to processing efforts become interesting. The second study employed product designs (bikes, chairs, and lamps) as stimuli and a 2 (fluency: low, high) × 2 (processing style: automatic, controlled) × 3 (product type: bike, chair, lamp) experimental design to examine pleasure and interest as mediators of the relationship between stimulus fluency and design attractiveness. With respect to lamps and chairs, the results suggest that the effect of stimulus fluency on attractiveness is fully mediated by aesthetic pleasure, especially in the automatic processing style. Conversely, disfluent product designs can enhance design attractiveness judgments due to interest when a controlled processing style is adopted.

## Introduction

The existing research on aesthetic preferences has long established that aesthetic pleasure and interest are two distinct positive aesthetic responses (Berlyne, 1971). However, most empirical research considers only very broad aesthetic preference judgments, such as aesthetic liking or attractiveness, to capture participants’ aesthetic response. This results in some fundamental contradictions in the literature. In particular, many studies find that aesthetic preferences are triggered by the fluency with which a perceiver can process an object (for a review see: Reber et al., 2004). Another body of research, however, links aesthetic preferences to difficult-to-process stimulus characteristics, which challenges the notion that fluent processing triggers liking. For instance, it has been shown that the aesthetic liking of a design is positively influenced by its complexity, a stimulus characteristic that impedes the ease of processing (Landwehr et al., 2011). Other research has found that stimulus novelty, which is a stimulus characteristic that makes processing less fluent, is related to aesthetic preferences (Hekkert et al., 2003).

The recently introduced Pleasure-Interest Model of Aesthetic Liking (PIA Model; Graf and Landwehr, 2015) can resolve these contradictory preference patterns by suggesting that liking and attractiveness may be overly vague constructs to the study of aesthetic preferences and that it is therefore necessary to distinguish between pleasure-based liking and interest-based liking to understand the formation process of aesthetic liking and attractiveness. Because the literature on aesthetic preferences treats aesthetic liking and attractiveness judgments as equivalent concepts (e.g., Labroo and Pocheptsova, 2016), we use only the broader term, aesthetic liking, throughout the theoretical portion of this paper to simplify the reading. To explain the formation of the involved constructs, the PIA Model distinguishes between stimulus-driven automatic processing and perceiver-driven controlled processing. It further assumes that fluent automatic processing triggers aesthetic pleasure and that the reduction of disfluency during controlled processing elicits aesthetic interest. Given these considerations, this research investigates the processes underlying aesthetic liking based on the formation of pleasure and interest and the relation of these two positive aesthetic responses to processing dynamics. Based on the classification of different possible perspectives on the psychology of aesthetics suggested by Jacobsen (2006), the current research focuses on the role of the “mind” in producing aesthetic responses. Accordingly, this research makes at least three substantial contributions to the literature on the mental perspective on aesthetic processing. First, this research is the first to empirically investigate the formation of pleasure and interest based on a coherent theoretical mechanism, namely, processing dynamics. In this regard, we provide empirical evidence that pleasure is driven by a gut-level fluency experience, whereas interest is triggered by a cognitive-driven disfluency reduction experience. Second, on a more general level, we provide empirical evidence for the main predictions of the PIA Model, thus promoting the notion of a dual process perspective on fluency-based aesthetics. Finally, we extend the core version of the PIA Model by investigating the link between pleasure and interest to an overall liking judgment.

We structure the remainder of the article as follows. First, in the theoretical section, we briefly review the existing work on the formation of pleasure and interest and the integration of these two responses into the PIA Model. In the empirical section, we report the results of two studies. Study 1 demonstrates that the effect of picture fluency on pleasure is mediated by a gut-level fluency experience and that the effect of picture fluency on interest is mediated by a disfluency reduction experience that is negatively related to picture fluency and contingent on a controlled processing style. Study 2 reveals that pleasure, as well as interest, mediate the relationship between design typicality and design attractiveness, and that these mediation effects are moderated by a perceiver’s processing style. In addition, the results show that pleasure is the more important mediator of the effect of design typicality. Finally, we conclude with a general discussion of our findings.

## The two Routes to Aesthetic Liking: Pleasure and Interest

To date, one of the most parsimonious yet prominent theoretical accounts that has been proposed to explain the process underlying positive aesthetic responses is the fluency framework (Reber et al., 2004). This framework, which is well established both in the field of experimental aesthetics (Belke et al., 2010; Forster et al., 2015) and in applied fields of research, such as product design (Landwehr et al., 2011, 2013), can be summarized as follows. First, depending on an object’s visual properties and a perceiver’s past experience with the object, processing of the object can be more or less fluent. Second, the experience of processing fluency elicits a gut-level affective reaction. Finally, provided that the perceiver does not attribute this positive affective reaction to a different source, he or she will draw on this reaction when making an aesthetic evaluation of the object, which then leads to a more positive evaluation. Importantly, the positive aesthetic response, which is assumed to enhance the evaluation of the object, is theoretically conceived as aesthetic pleasure, i.e., a “pleasurable subjective experience that is directed toward an object and not mediated by intervening reasoning” (Reber et al., 2004, p. 365). However, although the fluency framework can explain various phenomena where easy-to-process stimulus characteristics are linked to aesthetic liking, it fails to explain why people are sometimes attracted by difficult-to-process stimulus characteristics such as novelty (Hekkert et al., 2003; Blijlevens et al., 2012) or visual complexity (Landwehr et al., 2011).

In order to integrate preferences for difficult-to-process stimulus characteristics into a fluency account, Reber et al. (2004) propose the operation of an additional evaluative process based on conceptual fluency. More specifically, they argue that low perceptual fluency, which refers primarily to the difficulty of identifying the physical identity of a stimulus, can be compensated by high conceptual fluency, which reflects the ease of stimulus meaning assignment. In a similar vein, Belke et al. (2010) propose that “higher-order” cognitive operations on the level of meaning assignment can positively affect the evaluation of disfluent stimuli. Furthermore, Muth and Carbon (2013) have proposed that perceptual insight during elaboration, also referred to as moments of “aha!”, can increase peoples’ evaluation of initially disfluent stimuli.

Even though these approaches do provide an explanation of why people also like disfluent stimuli, they do not explicitly specify the necessary preconditions that make people process stimuli in a way that disfluency can become aesthetically liked. Moreover, they also do not specify whether and how exactly the processes that form the aesthetic liking judgment differ between fluent and disfluent stimuli. A refined view on these open theoretical issues is offered by the PIA Model (Graf and Landwehr, 2015), which adopts some of the core ideas of these earlier approaches but additionally provides a coherent theoretical account of *when* and *why* fluent versus disfluent stimuli lead to aesthetic liking. In particular, the PIA Model differentiates between pleasure-based and interest-based aesthetic liking and explicitly specifies the processing dynamics of these two routes to aesthetic liking. In addition, the PIA Model also considers the conditions under which the one or the other route takes effect. According to the model, aesthetic pleasure and interest can be distinguished based on distinct processing dynamics associated with distinct processing styles. Essentially, the model, which builds on the duality of mental processes as postulated in social psychology (Strack and Deutsch, 2004), proposes that people can process an aesthetic object either through only automatic processing or through first automatic and then controlled processing. Importantly, controlled processing requires processing motivation, which is why automatic processing is the default type of aesthetic processing. The model further assumes that automatic processing is stimulus-driven and may elicit a pleasure-based positive aesthetic response. Controlled processing, by contrast, is perceiver-driven, and thus leads to an interest-based positive aesthetic response. The PIA Model suggests that processing dynamics may explain both the formation of pleasure and interest. More precisely, it is assumed that pleasure is triggered by an experience of fluency during automatic processing, while interest is driven by an experience of disfluency reduction during controlled processing. Interestingly, this duality of mental processes suggested by the PIA Model also reflects recent insights from a (neuro-)physiological approach to aesthetics [i.e., the role of the “body” following the classification of Jacobsen (2006)], which confirm a distinction between controlled and automatic processing levels (Höfel and Jacobsen, 2007) and a differentiation of corresponding memory systems (Jacobsen, 2010).

Against the backdrop of the presented theorizing, the present research empirically examines the formation of aesthetic liking in a two-step process. First, we examined the formation processes of pleasure and interest by predicting that the processing dynamics underlying the relationship between stimulus fluency and pleasure are a gut-level fluency experience, while the dynamics underlying the relationship between stimulus fluency and interest are a cognitive-driven disfluency reduction experience. To test this, we exposed participants to three levels of stimulus fluency, and we manipulated perceiver-processing style to investigate how the dynamics of processing mediate the effect of these manipulations on pleasure versus interest (Study 1). In a second step (Study 2), we went beyond the tenets of the PIA Model by examining pleasure and interest as mediators of the relationship between stimulus fluency and an aesthetic liking response, which allowed for a complete picture of the mechanisms underlying global aesthetic judgments. Importantly, although the PIA Model construes both pleasure and interest as positive aesthetic responses, it does not explicitly theorize how these two responses are integrated into an overarching judgment of liking. For the present research, we predict that both pleasure and interest mediate the relationship between stimulus fluency and the aesthetic liking response. Furthermore, we expect that pleasure has a stronger influence than interest because the overall aesthetic experience is usually conceptualized as an affective judgment (Berlyne, 1971). Accordingly, the aesthetic liking response should align more closely with pleasure than with interest.

## Study 1

Study 1 was designed to investigate whether the effect of stimulus fluency on pleasure is mediated by a gut-level experience of fluency and whether the effect of stimulus fluency on interest is mediated by a cognitively driven disfluency reduction experience.

### Materials and Methods

#### Data Collection and Participants

Data collection took place in February 2014. Participants were recruited via Amazon MTurk and received a compensation of $0.45 for participating in the 11-min (approximate) online experiment. Importantly, because we are interested in “pure” aesthetic responses to visual stimuli, our participants do not need any specific expertise in the art-historical (or design-historical) context of the stimuli. Therefore, MTurk participants, who are demographically highly diverse (Buhrmester et al., 2011) and representative of the US population with regard to gender, race, age, and education (Paolacci et al., 2010) are highly suitable for the purpose of our studies. In addition, research suggests that the general data quality obtained with MTurk workers is high (e.g., Paolacci et al., 2010; Buhrmester et al., 2011; Sprouse, 2011; Paolacci and Chandler, 2014; Hauser and Schwarz, 2016). Both studies were conducted in accordance with the Declaration of Helsinki (World Medical Association [WMA], 2013). Participants provided informed consent, were debriefed at the end of the study, and had the opportunity to leave comments and/or contact the principal researcher. Ethical approval was not sought because it was assumed the research would not create distress or harm, and it consisted of anonymous questionnaires only (American Psychological Association [APA], 2002). A total of *N* = 424 subjects from the US completed in Study 1 (53% = female, *M*_age_ = 28), and all subjects were included in the analyses. Analyses were run after the final sample size had been collected.

#### Design and Stimuli

**FIGURE 1:**
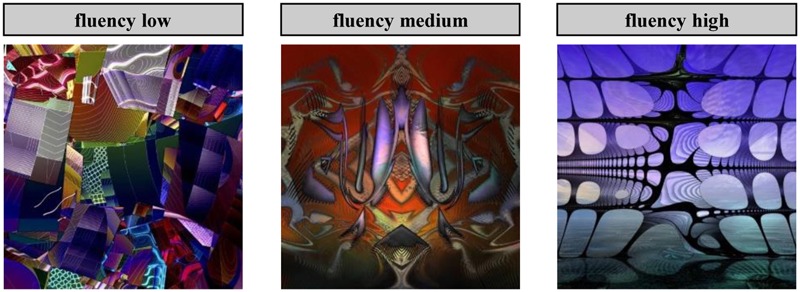
**Sample stimuli of Study 1.** Pictures from http://sanbase.com/gallery. html © 2016 San Base. All rights reserved. Used by permission.

The experimental design was a 3 × 2 × 2 mixed factorial design, where the first factor was a within-subjects manipulation of stimulus fluency (low, medium, and high), the second factor was a between-subjects manipulation of processing style (automatic, controlled), and the third factor was a between-subjects manipulation of the measured aesthetic response (pleasure, interest). Based on a prestudy with 904 participants, stimulus fluency was operationalized using colored abstract art pictures (generative digital art from a single artist) with varying fluency levels. Specifically, using an MTurk sample, we presented participants a random subset of 20 pictures drawn from a total of 458 pictures and asked them to indicate their subjective fluency experience for each picture (each picture was rated by at least 34 participants). Subjective fluency experience was measured using a three-item questionnaire with a visual analog scale of 101 continuous increments and the following labels: “The process of thinking about this picture … (1) is difficult for me/comes naturally to me, (2) is exhausting for me/is easy for me, (3) I perceive to be sluggish/I perceive to be smooth.” Psychometric analysis confirmed a reliable measurement of subjective fluency (α = 0.978). We selected three of the most disfluent, three of the most fluent, and three medium fluent pictures as stimuli for the present study. Accordingly, our final stimulus set (see **Figure 1**) consisted of nine pictures and represented three fluency levels (low, medium, and high) and three operationalizations per fluency level (low fluency pictures: *M* = 36.72, medium fluency pictures: *M* = 56.16, high fluency pictures: *M* = 74.84). Processing style was manipulated by giving participants different instructions. That is, participants were either asked to make a gut-level evaluation of how pleasing or interesting a picture is, or they were asked to focus intently on determining an appropriate title for the picture before making their evaluation. Importantly, the quality of the generated title was not evaluated and no feedback regarding the quality of the title was provided to prevent unnecessary evaluative stress for the participants.

#### Procedure

For the purpose of getting a measure of disfluency reduction, the study consisted of three evaluation phases: in the first and the third phase participants evaluated the pictures’ fluency, and the difference of these scores was used as a measure of disfluency reduction. In the first phase, participants were presented with the pictures in random order and asked to make a gut-level evaluation of their fluency experience regarding each picture (i.e., subjective baseline fluency). We used the same three-item questionnaire of fluency with the same visual analog scale of 101 continuous increments as used in the pretest. Participants were then assigned to one of the four between-subjects conditions. In the automatic processing condition participants were asked to merely give a gut-level evaluation of the pictures. In the controlled processing condition, by contrast, participants were requested to first concentrate intently about an appropriate title for the picture, and then to provide the title by writing it down. Participants were asked to evaluate either the pleasantness or the interestingness of the pictures. Pleasure and interest were each measured based on established scales using two items and the visual analog scales of 101 points, and pictures were again presented randomly. The items for pleasure were taken from Turner and Silvia (2006) and were, “I perceive the picture to be … (1) displeasing/pleasing, (2) unenjoyable/enjoyable.” Interest was measured with two items adapted from Silvia (Silvia, 2005a,b). These items were, “I perceive the picture to be … (1) disinteresting/interesting, (2) boring/exciting.” As a manipulation check, we then measured, using a seven point three-item scale, the degree of intensity exhibited by the participants as they interacted with the pictures before they evaluated them for pleasure/interest. The questions included, “How thoroughly and intensively (1) did you contemplate the pictures? (2) did you think about the pictures? (3) did you engage yourself with the pictures before you evaluated them?” Response options included, “not at all thoroughly/intensively” and “very thoroughly/intensively.” In a final evaluation phase, participants were again exposed to the pictures, and we asked them to give a gut-level evaluation of the pictures based on their current level of experienced fluency (i.e., post-processing fluency evaluation). In a final step, we obtained several individual difference variables related to art (art education, art activities; Furnham and Chamorro-Premuzic, 2004) and data regarding participants’ demographics.

### Results and Discussion

In all subsequent analyses, we averaged the ratings for pleasure, interest, and fluency across the three operationalizations per fluency level. Because we propose that fundamentally different psychological processes underlie the formation of pleasure versus interest, we report our results separately for these two dependent variables. Because the results remain robust when including the individual difference variables related to art as control variables, we excluded them from the subsequent analysis for the reason of clarity.

#### Pleasure

The analysis was conducted in two steps. First, we tested the effects of the experimental manipulations for our manipulation checks on all dependent variables. Second, we built a moderated mediation model that depicted the underlying process that connects stimulus fluency with pleasure.

**FIGURE 2:**
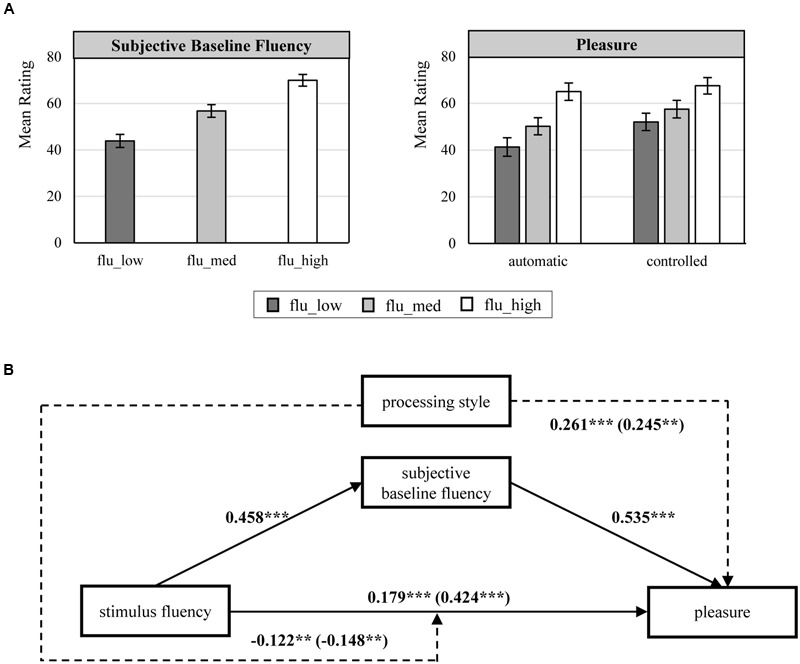
**(A)** Effects of experimental manipulations of picture fluency and processing style on subjective baseline fluency and pleasure. Error bars represent the 95% confidence interval of the means. **(B)** Moderated mediation analysis for the effect of stimulus fluency on pleasure mediated by subjective baseline fluency and moderated by processing style. Standardized beta-coefficients for all significant effects (*p* ≤ 0.05) are provided. All models were estimated using LMM and include a random effect for participants. Automatic processing = 0; controlled processing = 1; stimulus fluency_low = -1; stimulus fluency_med = 0; stimulus fluency_high = 1. The coefficients in brackets () refer to the model without the mediator “subjective fluency” ^∗∗^*p* < 0.01, ^∗∗∗^*p* < 0.001.

We first conducted an independent samples *t*-test that compared the mean scores of participants’ cognitive elaboration between processing style conditions. The results confirmed a significant difference in the mean elaboration scores for the automatic processing condition (*M* = 4.47, *SD* = 1.55) compared to the controlled processing condition (*M* = 5.92, *SD* = 1.04); *t*(213) = -8.085, *p* < 0.001. This clearly shows that the instruction to think of an appropriate title for the pictures successfully altered the extent to which participants elaborated the pictures, and it indicates that the type of processing varied between the two processing conditions. Next, we analyzed the ratings of participants’ subjective baseline fluency. As these ratings were collected before the experimental manipulation of processing style, we analyzed them across all participants in the pleasure condition. The results revealed that the pictures in the medium fluent condition were, on average, perceived as more fluent than the pictures in the disfluent condition and less fluent than the pictures in the high fluency condition (see **Figure 2A**). A repeated measures (RM)-ANOVA confirmed the statistical difference between these factor levels [*F*(1.77,377.93) = 193.672; *p* < 0.001; Greenhouse-Geisser 𝜀 = 0.883; $\eta_{p}^{2}$ = 0.475], and LSD *post hoc* contrasts between the three factor levels were all significant (*p* < 0.001). Notably, we adjusted the degrees of freedom (here and, whenever necessary, in the following analyses) using the Greenhouse-Geisser correction because Mauchly’s test indicated a violation of the sphericity assumption. Finally, we conducted a mixed-ANOVA on the pleasure ratings, including the fluency factor level as a within-subjects factor and processing style as a between-subjects factor. The results revealed a significant main effect of the fluency factor levels on the pleasure ratings [*F*(1.91,406.59) = 103.063; *p* < 0.001; Greenhouse-Geisser 𝜀 = 0.954], a significant main effect of processing motivation [*F*(1,213) = 9.980; *p* < 0.01], and a significant interaction effect of these two variables [*F*(1.91,406.59) = 4.526; *p* < 0.05; Greenhouse-Geisser 𝜀 = 0.954]. Upon examining the pattern of mean pleasure ratings, the results indicated that pleasure increases from the disfluent to the fluent pictures and that the profile of pleasure ratings across the three fluency factor levels differs between an automatic and a controlled processing style (see **Figure 2A**).

To test whether the effect of the experimental fluency manipulation on pleasure is mediated by subjective baseline fluency, we conducted a moderated mediation analysis, according to Muller et al. (2005), that allows the effect of the experimental fluency manipulation on subjective baseline fluency and the effect of subjective baseline fluency on pleasure to be moderated by processing style. Because our experimental design required repeated evaluations of the pictures, the error terms of the individual observations were not independent of each other. Thus, to analyze the data, we used linear mixed models (LMM) because this approach controls for the interdependence in the data structure by estimating fixed and random effects (Laird and Ware, 1982), and we relied on the lme() function of the nlme library of the software R (Pinheiro et al., 2015). All analyses were conducted based on the z-standardized values of fluency and pleasure. Furthermore, we used dummy coding for processing style (automatic processing = 0, controlled processing = 1), and we coded low stimulus fluency (flu_low = -1) as negative deviations and high stimulus fluency (flu_high = 1) as positive deviations from the medium fluent pictures (flu_med = 0). For reasons of clarity, we report the detailed results, i.e., standardized beta-coefficients and significance level, for all moderated mediation models investigated in Studies 1 and 2 within the figure corresponding to the analysis. The figures represent all significant paths (*p* ≤ 0.05) in the analyses, and the dashed paths represent significant effects that are, conceptually, not of primary interest. Consistent with our predictions, the positive effect of stimulus fluency on pleasure was mediated by the subjective baseline fluency experience (see **Figure 2B**). That is, the direct effect of stimulus fluency on pleasure when controlling for subjective baseline fluency was smaller than the total treatment effect of stimulus fluency on pleasure. In addition, stimulus fluency exhibited a significant effect on subjective baseline fluency, which in turn had a significant effect on pleasure. Although we included processing style as a potential moderator of the mediation effect, we found that processing style did not moderate the mediation effect. This suggests that the effect of stimulus fluency on pleasure was mediated by subjective baseline fluency regardless of processing style. To assess the statistical significance of this mediation, we used bootstrapping procedures by computing the indirect effect for 5,000 bootstrapped samples (MacKinnon et al., 2004). The 95% confidence interval of the bootstrapped indirect effect ranged from 0.191 to 0.304, which indicates that the indirect effect is statistically significant.

The negative interaction between processing style and stimulus fluency, as indicated by the left dashed path, suggests that the relationship between stimulus fluency and pleasure was attenuated when participants processed the picture on a controlled level. Presumably, this is because when a perceiver-picture interaction occurs during controlled processing, the immediate bottom-up effects of stimulus fluency are exhaustively channeled through the subjective fluency experience. Finally, the dashed right path from processing style to pleasure implies that participants rated the pictures as significantly more pleasant when they had processed them on a controlled processing level as opposed to an automatic processing level. This is most likely also due to the intensified interaction that occurs only during controlled processing.

Notably, we computed for all the estimated models the Intraclass Correlation Coefficients (ICCs) according to West et al. (2007). Specifically, for the estimated models the ICC is defined as the proportion of the total random variation in the responses that is due to the variance of the random intercept per participant and thus describes the homogeneity of subjects’ baseline evaluations of the pictures (i.e., the closer the ICC to zero, the higher the homogeneity of the baseline levels). For the pleasure ratings, the ICC is 0.276 in the model without baseline fluency as an additional predictor, and it is 0.174 in the full model controlling for baseline fluency. For the model predicting baseline fluency the ICC is 0.385.

#### Interest

**FIGURE 3:**
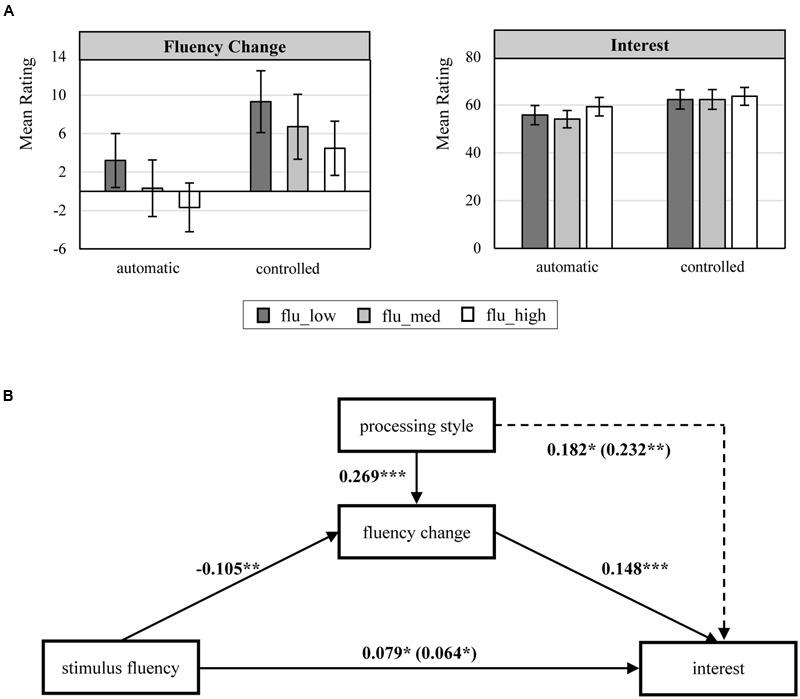
**(A)** Effects of experimental manipulations of picture fluency and processing style on fluency change and interest. Error bars represent the 95% confidence interval of the means. **(B)** Moderated mediation analysis for the effect of stimulus fluency on interest mediated by fluency change and moderated by processing style. Standardized beta-coefficients for all significant effects (*p* ≤ 0.05) are provided. All models were estimated using LMM and include a random effect for participants. Automatic processing = 0; controlled processing = 1; stimulus fluency_low = -1; stimulus fluency_med = 0; stimulus fluency_high = 1. The coefficients in brackets () refer to the model without the mediator “fluency change” ^∗^*p* < 0.05, ^∗∗^*p* < 0.01, ^∗∗∗^*p* < 0.001.

The analysis of the interest sample followed the same scheme as that of the pleasure sample. Again, participants interacted significantly more thoroughly with the pictures in the controlled processing condition (*M* = 6.02, *SD* = 1.01) than in the automatic processing condition [*M* = 4.83, *SD* = 1.61; *t*(207) = -6.241, *p* < 0.001], which indicates that type of processing varied between the two processing conditions as intended. As a manipulation check of the fluency manipulation, we analyzed the mean subjective baseline fluency ratings across the experimental fluency conditions (low fluency pictures: *M* = 43.82; medium fluency pictures: *M* = 56.76; high fluency pictures: *M* = 69.96) using an RM-ANOVA that compared the perceived subjective baseline fluency between the levels of manipulated fluency. The results [*F*(1.84,381.95) = 188.738; *p* < 0.001; $\eta_{p}^{2}$ = 0.476; Greenhouse-Geisser 𝜀 = 0.918] demonstrate that the fluency ratings differed significantly from each other and that all LSD *post hoc* contrasts were significant (*p* < 0.001). Next, we computed fluency change scores, i.e., disfluency reduction scores, as the difference between fluency evaluations before and after the processing and the evaluation phase of the pictures, such that a positive value indicates that disfluency reduction occurred during the processing of the picture. A mixed-ANOVA on these fluency change scores using the fluency factor levels as a within-subjects factor and processing motivation as a between-subjects factor found a significant main effect of the fluency manipulation [*F*(2,414) = 7.326; *p* < 0.01], a significant main effect of processing motivation [*F*(1,207) = 16.295; *p* < 0.001], and no significant interaction effect between these two variables [*F*(2,414) = 0.007; *p* = 0.993]. The pattern of the mean fluency change scores (see **Figure 3A**) indicated that the participants were able to reduce more disfluency when the stimulus fluency was lower. In addition, participants reduced a greater amount of disfluency in the controlled processing condition than in the automatic processing condition (see **Figure 3A**), which indicates that disfluency reduction is contingent on a controlled processing style. Finally, we conducted a mixed-ANOVA on the mean interest ratings that included fluency manipulation as a within-subjects factor and processing motivation as a between-subjects factor. The results of this model yielded no significant main effect of fluency [*F*(1.89,390.73) = 2.683; *p* = 0.073; Greenhouse-Geisser 𝜀 = 0.944] and no significant interaction effect between fluency and processing motivation [*F*(1.89,390.73) = 0.882; *p* = 0.409; Greenhouse-Geisser 𝜀 = 0.944]. The main effect of processing motivation, however, was significant [*F*(1,207) = 7.793; *p* < 0.01]. This lack of interest variation across fluency levels may be explained by the pattern of the fluency change scores. More precisely, as our theorizing predicts that the relationship between stimulus fluency and interest is mediated by disfluency reduction, the lack of variation in the total effect of stimulus fluency on interest may be explained by two opposing processes. On one hand, the affordance to reduce disfluency is, by definition, higher for disfluent stimuli, such that disfluent stimuli have a higher potential to become interesting due to successful disfluency reduction. On the other hand, the generic positive hedonic marking of fluent stimuli (Reber et al., 2004) may partially enter the interest judgment, which would produce a direct positive effect of fluency; because the indirect effect of disfluency reduction and the direct effect of fluency have opposite signs, they would cancel each other out.

To validate these considerations, we examined the cognitive dynamics underlying the relationship between stimulus fluency and interest. Further, we conducted a moderated mediation analysis that examined whether the effect of stimulus fluency on interest is mediated by fluency change, while allowing the potency of this mediating process to depend on participants’ processing style. We used the same LMM approach and variable coding scheme as we did for the analysis of pleasure, and we conducted our analysis based on the z-standardized ratings of fluency change and interest. The results (see **Figure 3B**) indicate that fluency change significantly affects interest and that the effect of stimulus fluency on interest increases when controlling for fluency change. This pattern of results indicates that fluency change suppresses the relationship between stimulus fluency and interest. In addition, the moderated mediation model revealed that fluency change is negatively related to stimulus fluency but positively related to a controlled processing style. Overall, these results explain why there is no considerable variation in the mean interest ratings across the experimental fluency conditions. That is, although stimulus fluency *per se* has a positive effect on interest, disfluency reduction, which is negatively associated with stimulus fluency, has a positive effect as well. Finally, we tested the significance of the indirect effect using bootstrapping with 5,000 resamples. The 95% confidence interval ranges from -0.034 to -0.003. Because this interval does not contain 0, the indirect effect is significant. Finally, we again computed the ICCs for the estimated models; for the models with interest as the dependent variable the ICC is 0.282 when fluency change is not accounted for and it is 0.292 when fluency change is included as a predictor. For the model with fluency change as the dependent variable the ICC is 0.156.

## Study 2

Whereas Study 1 investigated the relationship between stimulus fluency and pleasure versus interest, Study 2 took a broader perspective and examined pleasure and interest as mediators of the relationship between stimulus fluency and an overall aesthetic liking response. Moreover, Study 2 was also intended to attest to the generalizability and external validity of the examined effects. To this end, Study 2 examines the aesthetic appreciation of product designs instead of abstract art. Thereby, the present research provides practically useful information for applied aesthetic disciplines such as the management of product design (Landwehr, 2016) over and above presenting a mere theoretical examination. On a related note, Study 2 employed a more subtle, naturally occurring manipulation of processing style. Because it is more common to ask for design attractiveness in the product design literature (e.g., Crilly et al., 2004; Carbon and Leder, 2005), we adopted this as our measure of a global aesthetic (liking) response. Please note that attractiveness is one of the terms people intuitively associate with the aesthetics of objects (Jacobsen et al., 2004), which makes it an equally adequate dependent variable.

### Materials and Methods

#### Data Collection and Participants

Data for this study were collected in March 2016 on Amazon MTurk. Participants received a compensation of $0.55, and the study took approximately 8 min. A sample of *N* = 502 (50% = female, *M*_age_ = 36) participants from the US completed the study. All subjects were included in the analyses, and the analyses were run only after the final sample had been collected.

#### Design and Stimuli

The set-up of the study was a 2 (fluency: low, high) × 2 (processing style: automatic, controlled) × 3 (product type: bike, chair, lamp) experimental design, where fluency was randomly manipulated either within-subjects or between-subjects, processing style was manipulated within subjects, and product type was a between-subjects replication factor. Because design typicality is a particularly important determinant of peoples’ aesthetic response (Landwehr et al., 2011) and has been shown to affect fluency (Reber et al., 2004) it was used as an operationalization of fluency. Using two operationalizations per typicality level and product category, our final stimulus set consisted of 12 product designs. The stimuli included colored pictures of bikes, chairs, and lamps, all of which are freely available on the Internet. Picture selection and classification as more or less typical were conducted by one of the researchers of this study following short discussions with product design experts. In addition, we conducted a pretest with 160 participants on MTurk to validate that the designs differed in perceived typicality, which was assessed by the statement, “I perceive this product’s design to be…” and measured on a scale ranging from “not at all typical” to “very typical” (Blijlevens et al., 2012). The results of the RM-ANOVAS, which were conducted to compare the typicality ratings (average of the two operationalizations) across the two typicality levels for each product type revealed that all the mean typicality scores differed significantly from each other [bikes: *F*(1.74,67.95) = 327.282, *p* < 0.001, Greenhouse-Geisser 𝜀 = 0.871, $\eta_{p}^{2}$ = 0.849; chairs: *F*(1.66,64.61) = 427.630, *p* < 0.001, Greenhouse-Geisser 𝜀 = 0.860, $\eta_{p}^{2}$ = 0.916; lamps: *F*(1.40,54.49) = 263.968, *p* < 0.001, Greenhouse-Geisser 𝜀 = 0.699, $\eta_{p}^{2}$ = 0.871].

**FIGURE 4:**
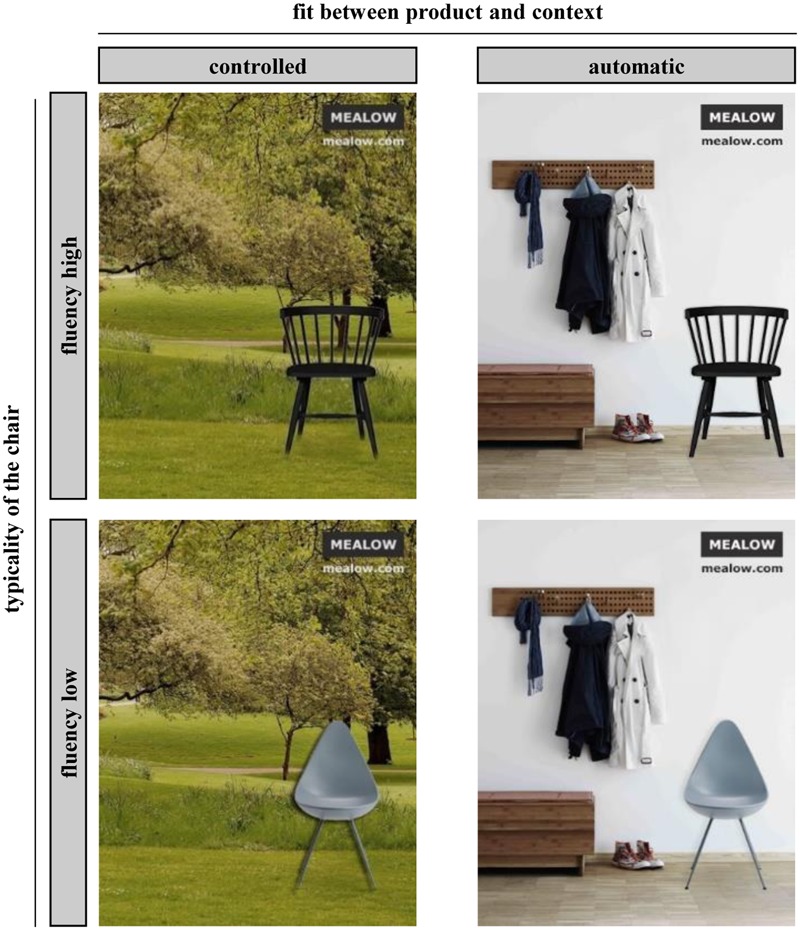
**Sample stimuli of Study 2**.

Participant processing style was manipulated by varying the usualness of the visual context in which a design was presented. This operationalization of processing style was based on the assumption that compared to a usual presentation context, an unusual presentation context would irritate the perceiver by attracting attention due to its unusualness and thus provoke an intent to better understand the stimulus. Notably, this idea of stimulus decontextualization as a means to induce more elaborate processing is similar to the notion of a ready-made in art, where an everyday object is separated from its usual context and put into an unusual representative context to evoke a more elaborative processing style that extends beyond simple object recognition (Gerger et al., 2014). For each product category, we created two different backgrounds depicting either a usage context that fit with the product or a usage context that did not fit with the product (see **Figure 4**). For instance, a chair was either depicted next to a wardrobe (fit context) or in a park (non-fit context). We framed our stimuli as advertisements by including a brand name and the brand’s web address (both the brand name and the web address were constant across all stimuli).

#### Procedure

At the onset of the study, participants were told that they would be shown various products of a fictitious brand within the same standard advertisement. Participants were then randomly assigned to one of the three product type conditions (between subjects: bike versus lamp versus chair). Each participant was presented two designs, one of which was presented in a fitting context and one presented in a non-fitting context. Importantly, the instructions between the two presentation context conditions were exactly the same; the only difference between the two conditions was the background picture surrounding the product stimuli. The two designs were randomly selected from among four stimuli per product category, but participants always evaluated two different designs, i.e., participants randomly viewed either one typical and one atypical design or two atypical/typical designs. First, we asked participants to make an overall attractiveness judgment of each design using the item, “Taking into account my overall feelings, thoughts, and impressions toward the bike/lamp/chair, I perceive the bike’s/lamp’s/chair’s design to be….” The response was assessed based on a scale of 101 continuous increments whose endpoints were unattractive (0)/attractive (101). The respondents then evaluated the interestingness and pleasantness of each design using the same measures as those used in Study 1. Finally, we assessed the participants’ expertise and interest in the respective product category, as well as their Centrality of Visual Product Aesthetics (Bloch et al., 2003).

### Results and Discussion

**FIGURE 5:**
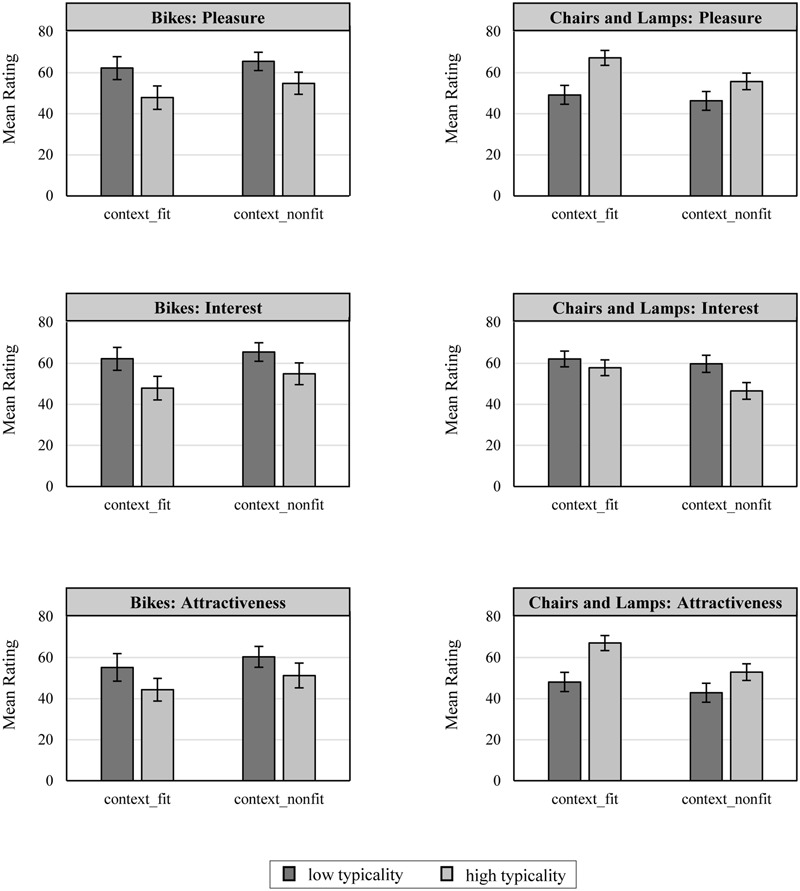
**Effects of experimental manipulations of design fluency and context type on pleasure, interest, and attractiveness.** In the left column, the results refer to bikes only, and the right column represents the results averaged across chairs and lamps. Error bars represent 95% confidence intervals of the means.

In a first instance, we averaged the ratings of pleasure, interest, and attractiveness across the two operationalizations per typicality level, and we analyzed the mean ratings of these scores across the experimental conditions per product category level. These initial analyses indicated that the typical bikes were consistently evaluated less favorably than the atypical bikes for all dependent variables, including attractiveness, pleasure, and interest, in both the context fit and the context non-fit condition (see **Figure 5**). This finding is inconsistent with the literature on product design attractiveness, which usually finds spontaneous preferences for typical designs (e.g., Halberstadt, 2006; Landwehr et al., 2011). Moreover, because the typicality effect is not contingent on context manipulation, the strength of the typicality manipulation was apparently too extreme to leave any room for context modulation. These observations suggest that the evaluation of the bikes did not follow pure aesthetic considerations and that the employed stimuli were not adequate to answer the present research question. Therefore, we excluded the category of bikes from all subsequent analyses.

**FIGURE 6:**
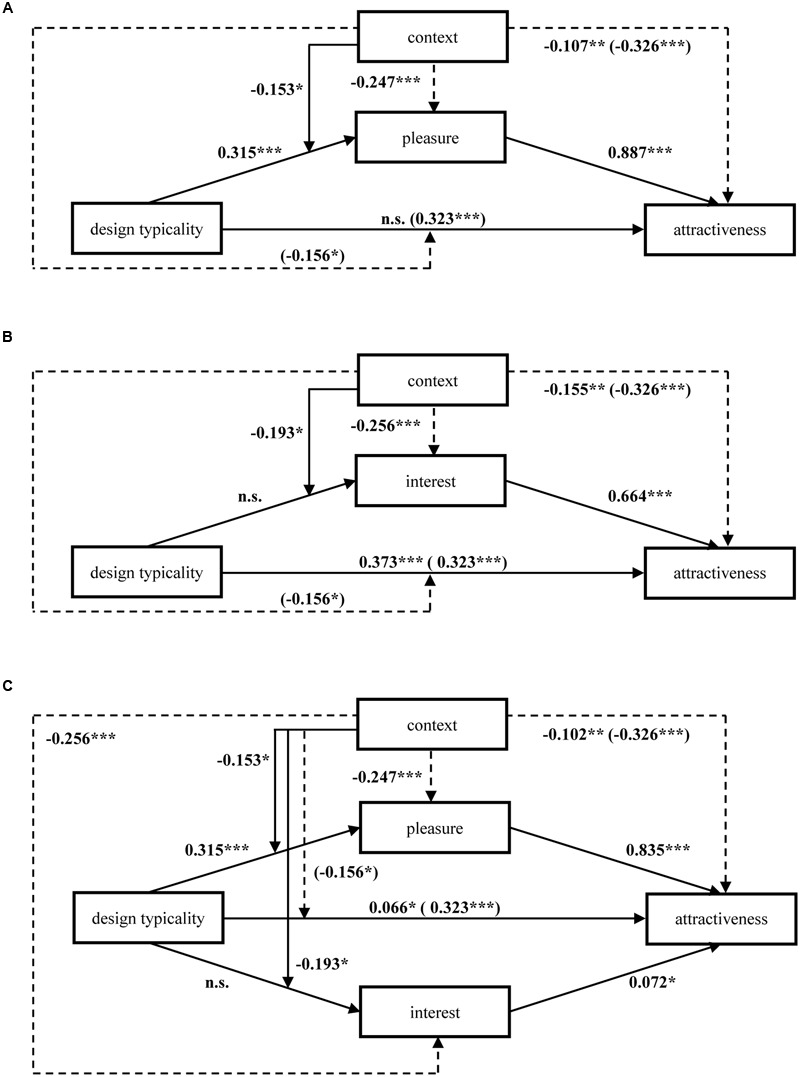
**Moderated mediation analyses.** The effect of design typicality on attractiveness mediated by pleasure **(A)** or interest **(B)** and moderated by context and the effect of design typicality mediated by both pleasure and interest and moderated by context **(C)**. Standardized beta-coefficients for all significant effects (*p* ≤ 0.05) are provided. All models were estimated using LMM and include a random effect for participants. Context fit = 0; context non-fit = 1; design typicality low = -1; design typicality high = 1. The coefficients in brackets () refer to the respective model without the mediator. ^∗^*p* < 0.05, ^∗∗^*p* < 0.01, ^∗∗∗^*p* < 0.001.

Because the patterns of mean ratings for chairs and lamps were highly similar to each other, we averaged these ratings across the product type replication factor for subsequent analyses. To obtain a first impression of the general pattern of results, we followed an inference-by-eye approach (Cumming and Finch, 2005). The corresponding tests and results for statistical significance that accounted for the complexity of the mixed experimental design are reported as part of the subsequent moderated mediation model (see **Figure 6**). Based on the means and their 95%confidence intervals provided in **Figure 5**, we can infer that with respect to pleasure and attractiveness, typical designs are preferred over atypical designs. However, with respect to interest, the pattern reverses. That is, atypical designs are preferred over typical designs. Moreover, these main effects appear to be qualified by context manipulation, such that especially in the context fit condition, typical designs benefit with respect to pleasure and attractiveness. However, with respect to interest, atypical designs benefit especially in the context non-fit condition. As theoretically predicted, this suggests that context moderates the effect of design typicality on pleasure versus interest. Moreover, the mean design attractiveness ratings were similar to the pattern of pleasure ratings, indicating that design attractiveness may be driven, to a greater extent, by pleasure.

To prepare the subsequent analyses that more deeply examined the dynamics underlying design attractiveness, we z-transformed the attractiveness, pleasure, and interest ratings per product category to exclude between product category variance from the analyses. To examine whether the effect of design typicality on attractiveness was mediated by pleasure or interest and whether these mediating effects were moderated by the presentation context, we relied on the same LMM approach for moderated mediation analysis as that used in Study 1 (Muller et al., 2005). Analogous to Study 1, we used dummy coding for presentation context (context fit = 0, context non-fit = 1), and we coded low design typicality as -1 and high design typicality as 1. Again, because the results are robust when including product expertise and product interest as control variables, we do not include them in the subsequent analyses. Also, we checked the ICCs of the estimated models; the ICC for the dependent variable attractiveness ranges between 0.001 and 0.027. With regard to pleasure and interest, the ICC is 0.031 for pleasure and 0.098 for interest.

Regarding pleasure-based attractiveness, we found that the effect of design typicality on attractiveness was non-significant when including pleasure as a mediator, but design typicality had a significantly positive effect on attractiveness when pleasure was not controlled for (see **Figure 6A**). This indicates that pleasure fully mediates the relationship between design typicality and attractiveness. The results further showed that the mediation effect was moderated by presentation context, such that the effect of typicality on pleasure was less pronounced when the design was presented in a non-fitting context. This suggests that design typicality has a reduced impact on pleasure once people engage in controlled instead of automatic processing. Importantly, pleasure affects liking regardless of processing style. The dashed paths, which are not directly relevant to our theorizing, indicate that the context manipulation has a negative effect on attractiveness and moderates the total effect of design typicality on attractiveness. These effects indicate that typical designs were liked less in a non-fitting context than in a fitting context. To assess whether the conditional indirect effects are statistically significant, we followed the bootstrap approach for moderated mediation proposed by Preacher et al. (2007). That is, we computed the bootstrapped confidence interval for the indirect effect conditional on both presentation context conditions. Based on 5,000 bootstrapped samples, the confidence interval of the indirect effect in the context fit condition is (0.193; 0.379), whereas in the context non-fit condition it is (0.048; 0.223). Thus, the indirect effect is significant in both presentation context conditions. Finally, we computed the bootstrapped index of moderated mediation (Hayes, 2015), which shows that the two indirect effects significantly differ in magnitude from each other (0.010; 0.293).

With respect to interest-based attractiveness (see **Figure 6B**), we found a suppressor effect such that interest suppressed the relationship between design typicality and attractiveness. More precisely, we found that the effect of design typicality increased when controlling for interest. Importantly, there was no effect of design typicality on interest but only an interaction effect between design typicality and context with respect to interest. This indicates that the suppression effect of interest takes effect only when the design is presented in a non-fitting context because only under this condition do atypical designs elicit interest. Presumably, this is because participants experienced only in the non-fitting context a sufficient amount of disfluency reduction to trigger interest. Again, the dashed paths, which show a highly similar pattern to pleasure-based attractiveness, suggest main effects of context manipulation on attractiveness and interest and, more importantly, an interaction effect due to which the total positive impact of typicality on attractiveness is reduced under controlled processing. Using the same bootstrapping approach to assess the moderated mediation effect which we employed for pleasure, the confidence interval in the context fit condition is (-0.103; 0.028) and (-0.255; -0.120) in the context non-fit condition. These results support our conclusion that the mediation effect takes effect only in the context non-fit condition but not in the context fit condition. The index of moderated mediation (Hayes, 2015) confirms that the two indirect paths differ significantly from each other (0.051; 0.255).

To examine the joint effect of pleasure and interest, we additionally estimated a model that included pleasure, interest, design typicality, context, and the interaction term between context and each of the other three predictors as the independent variables and attractiveness as the dependent variable. In conjunction with the models estimated before, the standardized beta-coefficients of this model (see **Figure 6C**) indicate that pleasure affects attractiveness to a greater extent than interest. For this model we also computed the confidence intervals for the bootstrapped conditional indirect effects. For pleasure, the confidence interval is (0.181; 0.362) in the context fit condition and (0.045; 0.212) in the non-fit condition. The index of moderated mediation (Hayes, 2015) is significant (0.001; 0.278). For interest, the confidence interval is (-0.036; 0.011) in the context fit condition and (-0.072; 0.022) in the non-fit condition. The index of moderated mediation (Hayes, 2015) is not significant (-0.011; 0.036). Hence, in this full model with pleasure and interest as mediators, only the indirect effect via pleasure is significant but not the indirect effect via interest. Finally, we re-estimated this final model but also included an interaction effect between pleasure and interest. The results remain robust, and the interaction effect between pleasure and interest with respect to attractiveness was significantly positive (*b* = 0.047, *p* < 0.01), thus indicating that the effects of pleasure and interest mutually reinforce each other.

## General Discussion

The present research investigates the processes underlying aesthetic liking and design attractiveness judgments based on two distinct positive aesthetic responses: pleasure and interest. Building on the core premise of the PIA Model (Graf and Landwehr, 2015), we propose that both pleasure and interest are triggered by processing dynamics but that the manifestation of this dynamic differs between the two responses. As expected, Study 1 indicates that the effect of stimulus fluency on pleasure is mediated by a gut-level fluency experience and that stimulus fluency and interest are related through a process of disfluency reduction. More specifically, we find that disfluency reduction suppresses the relationship between stimulus fluency and interest as disfluency reduction is negatively influenced by stimulus fluency. Study 2 finds that pleasure and interest mediate the effect of stimulus typicality on overall design attractiveness and that pleasure is a stronger predictor of attractiveness than interest. Importantly, interest is triggered only under controlled processing and is contingent on atypicality, while pleasure is triggered by typicality and amplified by automatic processing.

The above findings corroborate our proposition that aesthetic liking and its proxy design attractiveness are not unidimensional constructs. Rather, they are dual constructs that can be triggered by both pleasure and interest. Importantly, we find that pleasure and interest are inversely related to easy-to-process stimulus characteristics. Specifically, pleasure is positively related and interest is negatively related to easy-to-process stimulus characteristics. This dichotomy may provide an explanation for previously inconclusive findings regarding the relationship between fluency and liking. In addition, our results are in accordance with the main predictions of the PIA Model (Graf and Landwehr, 2015). More precisely, we find that aesthetic responses are a function of both processing dynamics and processing style. As indicated in Study 1, because pleasure is directly driven by the fluency that emanates from the stimulus, it does not require any controlled stimulus processing. By contrast, the experience of disfluency reduction, which drives interest, originates from the active engagement of the perceiver, which requires controlled processing.

Therefore, our findings indicate that the valence of aesthetic judgments critically depends on the perceiver’s processing style that interacts with the ease or difficulty of stimulus processing. With regard to future empirical research on aesthetic preferences, these findings strongly suggest the importance of assessing pleasure and interest rather than overall liking or to control for perceiver processing style.

In addition, future research should explore the relationship between pleasure and interest with respect to liking. That is, under what conditions do both pleasure and interest contribute to aesthetic liking? The PIA Model (Graf and Landwehr, 2015) remains rather vague at this point, actually suggesting that either pleasure or interest is triggered depending on the processing style. However, our empirical analysis suggests that when people are explicitly asked to indicate both pleasure and interest (as in Study 2), they report them as two distinct responses, both of which are related to aesthetic liking. This raises the question as to whether people usually experience both responses but that one is the more dominant response. Importantly, our finding that pleasure is a stronger predictor of liking may be related to the subtle manipulation of processing style, which did initiate controlled processing only to a minimal degree. Future research should thus replicate Study 2 using a stronger manipulation of processing style. On a more general level, future research may examine additional ways to manipulate peoples’ aesthetic processing style using manipulations that are more common in everyday life to examine the practical scope of our findings. The results of Study 2 show that our results are robust when using applied stimuli and a rather naturalistic way of manipulating processing style, which indicates that transferring our results to applied fields of research may be a fruitful approach to gain further insights on the nature of aesthetic experiences (see also Landwehr, 2016).

Interestingly, the ICCs of the estimated models are all rather low. This indicates that respondents’ baseline evaluations of the stimuli are highly homogeneous across respondents. In addition, we find that the ICC is considerably lower for the product design stimuli compared to the art stimuli. In fact, this divergence may reflect that aesthetic processing differs across content domains (Jacobsen, 2006; Höfel and Jacobsen, 2007), and it suggests that peoples’ basic aesthetic response to design is more homogeneous than their aesthetic response to abstract art. A limitation of Study 2 is that we did not find any effects for the product category of bikes, one of our between-subjects replication factors for stimulus fluency. Although it is surprising that the atypical bikes were consistently rated as more pleasant, interesting, and attractive, the consistent pattern suggests that our experimental manipulation of fluency was disrupted. One explanation may be that the more typical bikes did not meet peoples’ functional or ergonomic requirements; thus, the principle of hedonic dominance took effect (Chitturi et al., 2007). That is, because the functional requirements were not met, the bikes were not rated based on hedonic features, such as aesthetic appearance, but rather, were rated only with respect to functional features. This would also explain why the established positive effect of design typicality on attractiveness with respect to bikes (Halberstadt, 2006; Landwehr et al., 2011) was not present. Another limitation of our research is that we focused on the “mind” and did not directly examine processes of the “body” [following the classification of the psychology of aesthetics by Jacobsen (2006)]. With respect to future research, we see great potential in considering the discussed processing dynamics from a (neuro-)physiological perspective because the automatic and controlled effects of processing fluency should not only be reflected in a subjective experience but also in body processes (see for instance Höfel and Jacobsen, 2007; Jacobsen, 2010).

## Conclusion

Our research contributes to a holistic understanding of the formation process of aesthetic liking by providing empirical evidence of a dual route to aesthetic liking that is based on a single mechanism. As a result, this paper strengthens the construct of processing dynamics as a particularly important determinant of aesthetic appreciation.

## Ethics Statement

Both studies were conducted in accordance with the Declaration of Helsinki (World Medical Association [WMA], 2013). Participants provided informed consent, were debriefed at the end of the study, and had the opportunity to leave comments and/or contact the principal researcher. Ethical approval was not sought because it was assumed the research would not create distress or harm, and it consisted of anonymous questionnaires only (American Psychological Association [APA], 2002).

## Author Contributions

LG and JL conceptualized the studies reported in the manuscript. LG collected and analyzed the data of both studies; interpretation of the results was done by LG and JL together. Both LG and JL jointly wrote the manuscript.

## Conflict of Interest Statement

The authors declare that the research was conducted in the absence of any commercial or financial relationships that could be construed as a potential conflict of interest.
